# Epigenetic modulation with oral histone deacetylase (HDAC) inhibitor as a new treatment option in JIA

**DOI:** 10.1186/1546-0096-9-S1-P166

**Published:** 2011-09-14

**Authors:** J Vojinovic, A Furlan, N Damjanov, C D’Urzo, CA Dinarello

**Affiliations:** 1 Dept of Pediatric Rheumatology, University Clinic Center, Nis, Serbia; 2 Department of Medicine, University of Colorado Denver, Aurora, USA; 3 University of Padova, Padova, Italy; 4 Institute of Rheumatology, Belgrade, Serbia; 5 Italfarmaco, S.p.A, Cinisello Balsamo, Italy

## Background

Low concentrations of histone deacetylase (HDAC) inhibitors exhibit anti-inflammatory properties including animal models of arthritis [1]. Here we review possible mechanisms of action in arthritis and present results of HDAC inhibitor givinostat influence on cytokine network in patients with systemic JIA (SOJIA).

## Methods

Givinostat was orally administered for up to 12 weeks at a dose of 1.5 mg/kg/day in 17 SOJIA patients [2] with average disease duration of 59.53±49.16 months and duration of active disease of 14.19±24.41 months. Clinical assessment of disease activity was performed using ACR Pedi 30, 50 or 70 and a systemic feature score. Cytokines were measured in hole blood lysates using R&D Systems (Mosaic^TM^ ELISA) human cytokine panel.

## Results

Givinostat was safe and well tolerated with adverse events being mild or moderate, of short duration and self-limiting. Out of 17 patients who entered the study, ten completed the 12 weeks of treatment (58.8%). At week 4, the mean systemic feature score significantly decreased from 5.24 ± 0.37 to 2.59 ± 0.33 and the ACR Pedi 30, 50 or 70 improvement was 77.8%, 55.6% and 22.2%. At week 12, the ACR scores increased further to 77.8%, 77.8% and 66.7%, respectively. The most consistent finding was the reduction in the number of active joints and/or joints with limited range of motion. At week 4, in patients with baseline WBC of 12,000 or greater (N=11), there was a significant decrease (p<0.01) in the total WBC and absolute numbers of neutrophils (-30.6 and – 29.8 ∆% from baseline, respectively). Cytokines in whole blood lysates were elevated at baseline but after 2 and 4 weeks of givinostat, CD40L (p<0.05), IL 1α (p<0.001) and IFNγ normalised.

## Conclusion

HDAC inhibition by givinostat was safe and resulted in significant improvement of arthritis in SOJIA, as well as normalized cytokine and hematological parameters. We hypotheses possible use instead methotrexate to treat JIA after further investigations.
